# The relationships among self-control, psychological resilience, and digital addiction in college students: a meta-analytic structural equation modeling

**DOI:** 10.3389/fpsyg.2025.1650148

**Published:** 2025-09-17

**Authors:** Ziyang Zhao, Yaozhong Zhang, Yongqiang Ma, Azeem Obaid, Haidong Zhu

**Affiliations:** ^1^Normal College of Shihezi University, Xinjiang, China; ^2^Psychological Application Research Center, Shihezi University, Shihezi, China

**Keywords:** self-control, psychological resilience, digital addiction, meta-analysis, mediating effect

## Abstract

**Purpose:**

Digital addiction adversely affects the physical and mental well-being of college students. Comprehensive studies demonstrate that self-control has an inhibitory influence on digital addiction, with psychological resilience acting as a crucial mediating variable connecting self-control and digital addiction. Nevertheless, meta-analytic evidence supporting this mediating role is limited.

**Materials and methods:**

A meta-analysis employing structural equation modeling was conducted to examine the influence of self-control on digital addiction among college students. It encompassed 84 research studies and a total sample of 60,966 participants.

**Results:**

The findings indicate that psychological resilience modulates the connection between self-control and digital addiction, whereas self-control significantly influences digital addiction. This data suggests that when individuals exert considerable self-control to manage scholastic constraints or social responsibilities, their psychological resources drastically deplete, resulting in heightened digital media consumption. Moreover, self-regulated behaviors bolster psychological resilience by fostering a collection of good experiences, thereby diminishing escapist reliance on digital media.

**Conclusion:**

These findings elucidate the mediating process between self-control and digital addiction in college students, offering new intervention options and enhancing research on restricted self-control theory.

**Systematic review registration:**

https://doi.org/10.17605/OSF.IO/DUZQM.

## Introduction

The pervasive use of digital technology has rendered digital addiction a critical global public health concern. College students, identified as “digital natives,” are confronting the significant issue of internet addiction. A 2023 research study by the China Internet Network Information Center indicates a rising prevalence of internet addiction among Chinese adolescents, necessitating urgent measures for prevention and treatment. College students possess significant autonomy in their time, encounter numerous academic and social constraints, and are extensively engaged in the digital realm, making them more susceptible to the pitfalls of excessive digital product use, including social media and online gaming. Extensive research indicates that excessive engagement with digital products, including social media and online gaming, contributes to weight gain, mood disorders, and sleep disturbances (Duran and Alemdar, 2023; Lu et al., 2023; Ma et al., 2023), while also diminishing attention span and academic performance (Li et al., 2022; Zhang et al., 2024), thereby adversely affecting both mental and physical health, as well as academic progress. Self-control, defined as the fundamental capacity to suppress urges, manage behavior, and attain long-term objectives (Sjåstad and Baumeister, 2018), has been demonstrated to mitigate the risk of internet addiction significantly (Zhou and Zhou, 2017). Psychological resilience is an individual’s dynamic capacity to sustain psychological equilibrium and adjust favorably in the face of adversity (Wu et al., 2025), which aids college students in managing challenges and surmounting difficulties in the digital landscape (Hodge and Johnson, 2020). Prior research has individually investigated the correlations between self-control and psychological resilience, in addition to digital addiction. Discrepancies in participant groups, measurement instruments, study methodology, and geographical dispersion have resulted in inconsistent conclusions. This mismatch may be affected by cultural values. Chinese culture promotes an emphasis on long-term objectives, which may diminish the risk of digital addiction. In contrast, countries under different cultural frameworks frequently prioritize short-term objectives, thus heightening the danger of digital addiction (Li et al., 2021). Nonetheless, current research has primarily concentrated on variable connections within a singular cultural setting, failing to systematically investigate cultural influences. This study utilizes meta-analytic structural equation modeling to objectively analyze the links among self-control, psychological resilience, and digital addiction in university students from various cultural origins. It carefully examines model variations across cultural contexts to enhance our comprehension of these interconnected aspects.

### Self-control and psychological resilience

Studies indicate a substantial positive correlation between self-control and psychological resiliency. Individuals with elevated self-control can efficiently leverage social support resources, mitigate emotional variability, and consequently augment psychological resilience (Cai et al., 2022). College students exhibiting elevated self-control can proficiently inhibit the desire for immediate fulfillment and implement techniques aligned with their long-term objectives (Milyavskaya and Inzlicht, 2017). This fundamental regulation capacity allows people to employ more constructive coping mechanisms in response to challenges, thus augmenting their psychological resilience. Self-control allows college students to uphold their dedication to long-term value objectives, including academic success and the preservation of interpersonal connections. In the presence of distractions and temporary setbacks, persistence in goal pursuit diminishes negative thoughts like self-doubt, and, more crucially, the successful experiences gained from overcoming obstacles and achieving objectives substantially bolster an individual’s self-efficacy (Bao et al., 2024). Self-efficacy, characterized as an individual’s confidence in their coping abilities, constitutes the fundamental cognitive basis of psychological resilience (Schwarzer and Warner, 2013) and establishes a robust belief system that underpins the cultivation of total psychological resilience. Studies indicate that enhancing self-control can facilitate greater psychological resilience in college students under academic strain (Li Y. et al., 2024; Wang, 2025). Consequently, this research posits Hypothesis 1: Self-control exerts a direct beneficial influence on psychological resilience.

### Self-control and digital addiction

Digital addiction denotes obsessive conduct arising from the excessive utilization of electronic gadgets, encompassing internet addiction, gaming addiction, and smartphone addiction. This addictive behavior can adversely affect an individual’s physical and mental development (Ali, 2018; Almourad et al., 2020; Vadlin et al., 2015). The design features of digital technology settings closely correspond with persons’ inherent inclination for rapid pleasure (Turel and Qahri-Saremi, 2016), therefore presenting a continual challenge to individuals’ self-control resources. The Limited Strength Model of Self-Control asserts that self-control, regarded as a finite psychological resource, is essential for suppressing impulsive responses, managing automatic behaviors, and attaining long-term objectives (Baumeister et al., 2007). Individuals with low self-control frequently encounter heightened difficulties; they may possess fewer beginning resources, experience more rapid depletion of these resources when confronted with temptation, and lack efficient resource management skills (Brevers and Turel, 2019). Consequently, they are exceedingly vulnerable to resource exhaustion, resulting in unregulated consumption of digital media. Conversely, people possessing high self-control effectively manage media consumption through strategic resource allocation (Xian et al., 2019). A multitude of empirical investigations regarding smartphone dependence, online gaming addiction, and excessive social media usage have repeatedly demonstrated a negative correlation between self-control and digital addiction (Koç et al., 2023; Sun et al., 2025; Wang, 2025). This relationship is seen at the behavioral level as follows: Individuals with diminished self-control demonstrate a heightened attention bias toward digital stimuli, engage in more impulsive decision-making, and possess compromised fundamental behavioral regulation skills. Despite acknowledging the detrimental effects of excessive use, people find it challenging to successfully terminate or regulate their consumption patterns (Han et al., 2017). This study proposes Hypothesis 2: Self-control exerts a direct detrimental effect on digital addiction.

### Psychological resilience and digital addiction

Psychological resilience enables individuals to sustain stable psychological functioning amid stress, setbacks, or adversity, facilitating adaptive adaptation and recovery through the effective mobilization of internal and external resources (Feng et al., 2022). Research indicates a significant negative correlation between psychological resilience and digital addiction among college students, suggesting that higher psychological resilience enables more effective coping with real-world stress and diminishes the inclination to evade problems through digital media (Wang, 2025). This correlation arises from the observation that individuals possessing high psychological resilience are more adept at managing real-life stressors due to their enhanced internal adaptive capacities and external resource utilization, consequently diminishing their inclination to engage in escapist coping strategies through excessive digital media consumption.

Individuals exhibiting elevated psychological resilience are more adept at managing and adapting to negative emotions elicited by stress, thereby diminishing their dependence on digital media (Wu, 2018; Zhang and Zhang, 2016), and instead embracing proactive problem-solving strategies or pursuing tangible support in the real world. Individuals possessing robust psychological resilience are more inclined to employ proactive problem-solving strategies, diminish their propensity for immersion in the virtual realm, and consequently lessen their excessive concentration on digital content (R. Li et al., 2024) Simultaneously, familial support and interpersonal assistance, as fundamental components of psychological resilience, can enhance tangible social connections, diminish individuals’ dependence on digital social media as a replacement, and establish a protective mechanism against addiction (Öztürk and Kundakçı, 2021; Zhang and Zhang, 2016). College students experiencing various academic and social pressures, particularly those with diminished psychological resilience, are more inclined to engage in excessive mobile phone or internet usage to attain instant gratification and temporary relief from stress, owing to insufficient coping mechanisms. This elevates the likelihood of developing digital addiction. Consequently, in light of the preceding discourse, Hypothesis 3 may be articulated: Psychological resilience adversely affects digital addiction.

### The mediating role of psychological resilience

The idea of limited self-control asserts that self-control, as a finite cognitive resource, is essential for suppressing immediate desires and sustaining long-term goal orientation (Baumeister et al., 2007). Elevated self-control allows individuals to regulate and preserve this resource more efficiently, minimizing superfluous use or exhaustion in resisting digital enticements. The efficient management of psychological resources establishes a basis for individuals to utilize internal assets and incorporate external support systems in times of stress, therefore fostering the development and improvement of psychological resilience (Hodge and Johnson, 2020). Augmented psychological resilience, characterized by its fundamental abilities in emotional regulation, problem-solving orientation, and the mobilization of social support, equips individuals with more robust buffering mechanisms and adaptive strategies to manage immediate temptations in digital contexts, thereby diminishing the likelihood of succumbing to escapist usage and addiction. College students exhibiting elevated self-control can regulate digital media consumption via strategic resource distribution (Milyavskaya and Inzlicht, 2017). The successful experiences of self-regulation, together with the sensation of efficacy gained from resisting temptations, are vital for the formation and strengthening of psychological resilience (Cai et al., 2022). Individuals with robust self-control are more inclined to employ adaptive coping mechanisms, such as actively addressing difficulties, when confronted with scholastic pressure or social disappointments, rather than seeking emotional refuge in digital media (Zhang and Zhang, 2016). This enduring and effective adaptive coping strategy not only diminishes escapist behaviors but, more significantly, strengthens an individual’s psychological resilience by fostering a collection of successful experiences in overcoming challenges. This enhances an individual’s capacity to manage stress and temptation, establishing a virtuous protective cycle that diminishes the danger of digital addiction more sustainably and effectively (Wang, 2025). Consequently, we propose Hypothesis 4: Psychological resilience influences the influence of self-control on digital addiction.

### Previous meta-analysis

Prior meta-analyses have individually investigated the correlation among self-control, psychological resilience, and digital addiction. Numerous meta-analyses have consistently identified a negative correlation between self-control and behaviors associated with digital addiction, including internet addiction, mobile phone addiction, and excessive social media use (Ding et al., 2022; Hu et al., 2025; Ji et al., 2022; Koo and Kwon, 2014; Li et al., 2021; Ji and Son, 2024; Yeun and Han, 2016). A meta-analysis examining the association between psychological resilience and digital addiction reveals a strong negative correlation between the two (Hidalgo-Fuentes et al., 2023; Lyu et al., 2024). While these studies offer robust evidence for the correlation between self-control, psychological resilience, and digital addiction, the groups examined are at varying developmental stages. The interconnections among these three elements and the mediating function of psychological resilience remain inadequately clarified, necessitating additional research to investigate these matters more comprehensively.

### Current research

The correlation of self-control, psychological resilience, and digital addiction has been extensively examined. Nonetheless, owing to the variety of research methodologies and the absence of meta-analyses regarding the mediating function of psychological resilience between self-control and digital addiction, undertaking a thorough meta-analysis is highly significant. This study will utilize a meta-analytic structural equation model to perform a thorough and cohesive analysis of existing research, focusing on the mediating role of psychological resilience between self-control and digital addiction, exploring the relationships among self-control, psychological resilience, and digital addiction, and examining the moderating effects of moderator variables within this framework. We hypothesize that self-control positively influences psychological resilience (Hypothesis 1) and negatively impacts digital addiction (Hypothesis 2) and that psychological resilience negatively affects digital addiction (Hypothesis 3). Furthermore, we propose that psychological resilience mediates the relationship between self-control and digital addiction (Hypothesis 4).

## Method

### Literature search

This study employed a thorough and systematic literature search method, encompassing all languages within both Chinese and non-Chinese categories to guarantee the inclusiveness and representative of the data. The primary justification for using Chinese databases was the significant increase in recent years of study about college students’ digital addiction and associated psychological factors inside the Chinese academic domain. The data quality and sample variety were adequate to fulfill the criteria for sample size and data reliability in meta-analysis, hence validating the use of Chinese databases for the search. Chinese-language literature was obtained via the China National Knowledge Infrastructure (CNKI), Wanfang Database, and VIP Database. Non-Chinese literature included all language varieties and was accessed using databases such as ProQuest, EBSCO, and Web of Science to reduce the likelihood of omissions caused by language difficulties.

The key words of this study are psychological resilience (心理韧性、心理弹性、复原力), self-control (自我控制), digital addiction (数字成瘾), mobile phone addiction (手机成瘾 or 手机依赖), internet addiction (网络成瘾 or 网络依赖), social media addiction (社交媒体成瘾 or 社交媒体依赖). In addition, to ensure the completeness of the study and avoid any potential omissions, we will use the literature tracing method, which involves thoroughly exploring and tracking the reference lists in each piece of literature, as an important means of identifying and addressing any gaps. Furthermore, in this meta-analysis, we used the Boolean logic operator “OR” to combine terms with equivalent meanings. Subsequently, we used the operator “AND” to integrate these concepts. As of May 2025, a total of 4,623 articles were retrieved.

### Inclusion criteria

The literature utilized in this study was independently assessed by a minimum of two reviewers to ascertain its compliance with the specified criteria. This study employed the following criteria: (1) The article must be an empirical study, excluding reviews or meta-analyses; (2) the article must explicitly detail the measurement instruments employed; and (3) the article must examine the relationship among psychological resilience, self-control, and digital addiction, and must present *p*-values, Pearson correlation coefficients (r), or effect sizes convertible to r. Data derived from structural equation modeling, path analysis, regression analysis, or analogous methodologies are excluded; (4) samples from various studies must be independent; and (5) comprehensive measurement instruments or particular indicators must be employed for assessment.

### Coding rules

Data for each study was separately gathered by two researchers. In the event of a quarrel, the matter was addressed through consultations with psychological professionals. Each study was classified based on the subsequent criteria: (1) author of the study; (2) sample size; (3) correlation coefficient; (4) year of publication; (5) type of article; (6) source of article sample; and (7) quality score of the article.

### Risk quality assessment

The quality assessment predominantly utilizes a modified Key Assessment Tool (Karimi et al., 2023), whereby projects receive a score of 1 or 0, culminating in a maximum of 8 points. Scoring was performed independently by two evaluators, with differences addressed through consultation, and a third party consulted if required. Articles were classified into high-scoring groups (score > 6) and low-scoring groups (score < 6) according to the final evaluation. The checklist predominantly encompasses the following elements: Are the study’s hypotheses, objectives, and goals explicitly articulated? Is the study design suitable for accomplishing these objectives? Is the study sample representative? Are the psychometric properties of the mediating and outcome variables documented? Are statistically valid data analysis methods employed? Does the study expressly forecast that alterations in the predictor or mediator variables precede modifications in the outcome variables? Are the primary conclusions of the study explicitly articulated? Does the study account for potential confounding variables?

## Data analysis

### Extraction and calculation of effect size

Meta-analytic structural equations are incapable of deriving various effect sizes from a single study. When a single study presents numerous effect sizes, the following guidelines are established: When a study presents impact sizes across various dimensions, the mean of these effect sizes is computed to provide a thorough evaluation (Li et al., 2025). This averaging strategy facilitates a thorough and representative evaluation of the study.

The TSSEM method, created by Cheung and Chan in 2005, combines the correlation matrix with fitting a model to the data using Structural Equation Modeling (SEM). Initially, a multivariate random effects model was employed to aggregate the correlation matrix by maximum likelihood (ML) estimation. The machine learning method demonstrates superior accuracy and effectively manages missing variables, yielding reasonably precise estimations in comparison to alternative methods (Baraldi and Enders, 2010; Jamshidian and Bentler, 1999). In the second step, Cheung and Chan proposed employing weighted least squares (WLS) for model fitting by incorporating the first-stage correlation matrix into the structural equation model. Oort and Jak contended that both phases of the TSSEM may be assessed using the ML method; hence, this approach is termed Maximum Likelihood Meta-analytic Structural Equation Modeling (ML MASEM) (Oort and Jak, 2016). The initial stage mirrors that of TSSEM, encompassing the heterogeneity assessment and the construction of the correlation matrix via the ML approach. The distinction lies in the model fitting during the second stage; in ML MASEM, a common RMODEL is applied to the observation matrix of all studies, with the RMODEL capable of adopting the structure of any SEM. This work employed a two-stage structural equation modeling (TSSEM) approach for analysis.

We analyzed this study using the meta SEM package in R 4.5.0, with “r” serving as the effect size. Initially, the correlation coefficient was incorporated, followed by the computation of Fisher’s z value for the mean. Upon completion of the calculations, Fisher’s z value was converted. After the calculation, Fisher’s z value was transformed into the correlation coefficient “r” (Cooper et al., 2019), and then assessed for publication bias utilizing funnel plots and *Egger’s* test.

This research evaluated the relevance of the random-effects model via heterogeneity assessments. The random-effects model allows for authentic variations in effect sizes across studies, based on the assumption that these effect sizes are randomly distributed among the studies. This model is suitable when researcher’s hypothesis that substantial heterogeneity is present among studies. The random-effects model considers both the variance within individual studies and the variance between studies, resulting in an estimate of the average effect size. This study conducted a systematic review of the literature, identifying factors such as sample characteristics, article quality, and article type that influence the relationship between self-control, psychological resilience, and digital addiction. Thus, the random-effects model was employed for estimation. The random-effects model’s applicability was evaluated using heterogeneity tests, focusing on the significance of Q-test results and the I^2^ value. A random-effects model was utilized when both the Q-test and I^2^ value surpassed 75%, whereas a fixed-effects model was applied if either was below 75% (Huedo-Medina et al., 2006).

This study utilized both a full mediation model and a partial mediation model in the mediation analysis. The comprehensive mediation model demonstrates that psychological resilience entirely mediates the influence of self-control on digital addiction, while the partial mediation model indicates that psychological resilience partially mediates this effect. Furthermore, to evaluate the suitability of the model fit, the study will employ numerous indicators. The initial assessment will utilize the chi-square value, where a value below 2 signifies a strong model fit, and a value below 5 denotes acceptable fit. Secondly, the CFI, AGFI, GFI, and TLI will be assessed, with values ranging from 0 to 1; a number closer to 1 is often indicative of a superior match. Ultimately, RMSEA and SRMR will be assessed using RMSEA. It is widely recognized that an RMSEA value below 0.01 signifies an excellent model fit (Sugawara and MacCallum, 1993), values between 0.01 and 0.05 indicate a good model fit, values between 0.05 and 0.08 suggest a moderate model fit (Browne and Cudeck, 1992), and values between 0.08 and 0.10 reflect an acceptable model fit (MacCallum et al., 1996). SRMR values below 0.05 indicate a strong fit, whereas values below 0.08 are deemed acceptable for model fit (Hu and Bentler, 1998).

## Results

### Study selection and characteristics

**Figure 1:**
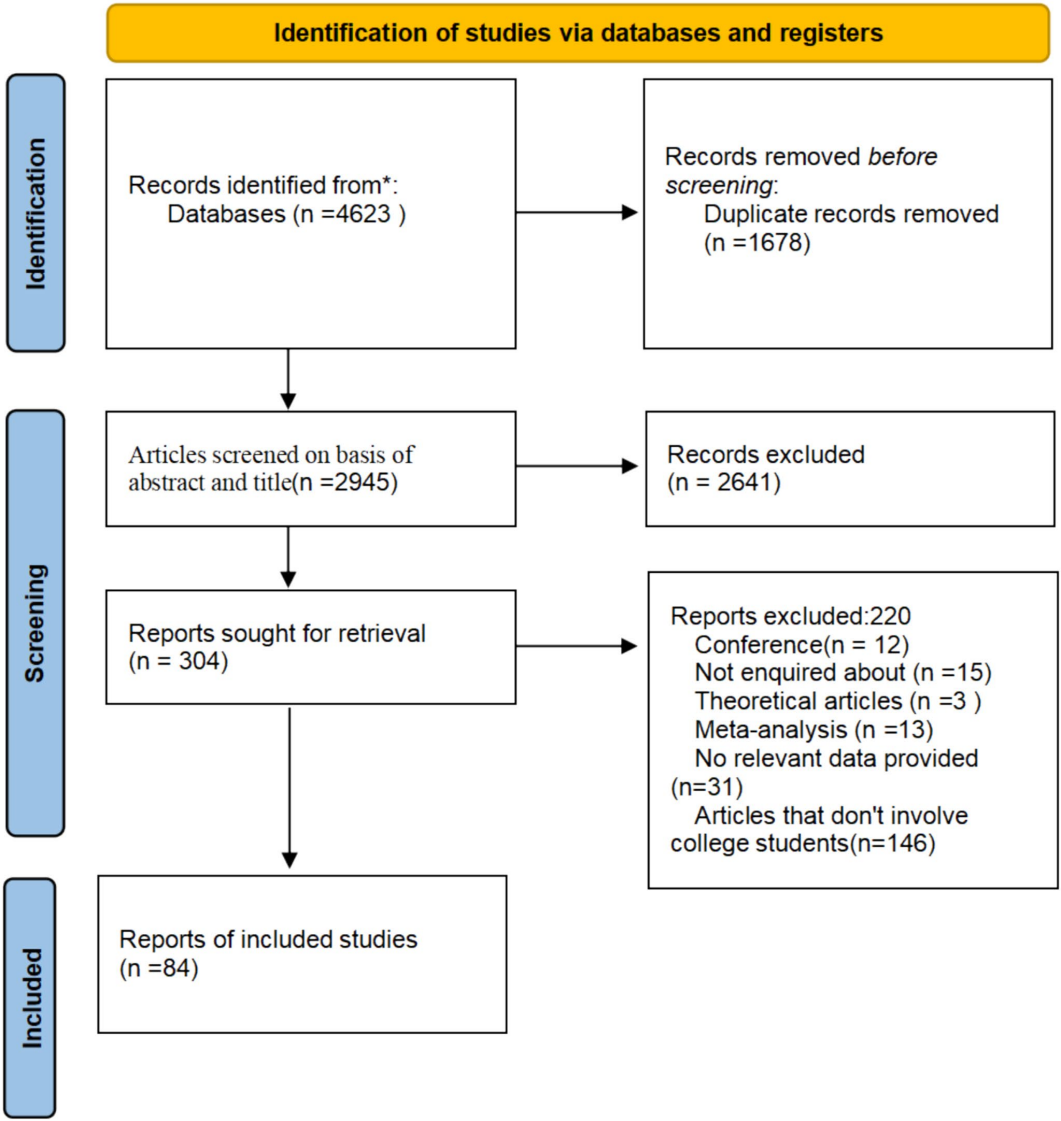
Screening flowchart.

After eliminating duplicates, the search yielded 4,623 articles, of which we retained 2,945. After scrutinizing the titles and abstracts, we ultimately selected 304 papers that contained relevant records. Two hundred twenty articles were excluded, as detailed in the PRISMA flowchart. The primary grounds for exclusion comprised 12 conference papers, 15 inaccessible publications, 3 theoretical works, 13 meta-analyses, 31 articles lacking pertinent data, and 146 articles not involving college students. A total of 84 articles were incorporated into the meta-analysis. Table 1 delineates the studies included. The combined sample size of this research was 60,966 participants, classified by design into thesis papers and journal articles, by quality into high-scoring and low-scoring groups, and by region into Chinese and non-Chinese areas (see Table 1; Figure 1).

**Table 1:** Included study characteristics.

Author	N	s_r	s_a	r_a	Year	Article	SCORE	SAMPLE
Cao Junrong	538	NA	−0.27	NA	2023	D	7	C
Chen Xuelian et al.	321	NA	−0.62	NA	2024	J	7	C
Du Zhihao et al.	855	NA	−0.57	NA	2023	J	7	C
Fan Cheng et al.	383	NA	−0.51	NA	2019	J	6	C
Feng Biao et al.	592	0.47	NA	NA	2022	J	7	C
Ge Dandan et al.	1,246	0.46	NA	NA	2021	J	7	C
Gong Yanping et al.	432	NA	NA	−0.433	2015	J	5	C
Guo Jingjing	304	0.487	NA	NA	2014	D	5	C
He Can et al.	453	NA	−0.468	NA	2012	J	6	C
Huang Lijuan et al.	433	NA	−0.46	NA	2021	J	8	C
Li Jiaojiao et al.	791	NA	−0.49	NA	2023	J	7	C
Li Shuang	294	NA	−0.545	NA	2024	J	6	C
Li Weiwei et al.	283	NA	−0.56	NA	2021	J	4	C
Li Xuan	339	NA	NA	−0.622	2024	J	6	C
Li Yue et al.	1,078	0.62	NA	NA	2024	J	7	C
Liang Feng et al.	708	NA	−0.62	NA	2020	J	7	C
Liu Lu et al.	479	NA	−0.64	NA	2023	J	5	C
Liu Ru et al.	1,083	NA	NA	−0.276	2023	J	7	C
Luo Suqi	2,505	NA	−0.494	NA	2024	D	7	C
Meng Dan et al.	312	NA	−0.576	NA	2019	J	3	C
Li Maoning et al.	913	NA	−0.468	NA	2024	J	6	C
Zuo Kuiyong et al.	663	NA	−0.536	NA	2023	J	6	C
Zhou Haili et al.	1,559	NA	−0.41	NA	2022	J	6	C
Zhou Enyuan et al.	1,313	NA	−0.414	NA	2017	J	6	C
Zheng Yanglei et al.	157	0.46	NA	NA	2022	J	6	C
Zhang Zhiying et al.	407	NA	−0.55	NA	2020	J	5	C
Zhang Yanli	301	NA	NA	−0.373	2020	D	6	C
Zhang Xian et al.	478	NA	−0.47	NA	2019	J	6	C
Zhang Bibibi et al.	351	NA	NA	−0.145	2016	J	5	C
Nie Yangang et al.	784	NA	−0.369	NA	2013	J	5	C
Peng Jie et al.	336	NA	−0.503	NA	2020	J	5	C
Shen Xiaowei	380	NA	−0.55	NA	2021	D	5	C
Shen Xiaowei	825	NA	−0.56	NA	2021	D	5	C
Shi Xiaoni	599	NA	−0.466	NA	2019	D	6	C
Wang Juan et al.	1986	NA	0.41	NA	2012	J	5	C
Wang Juanjuan et al.	355	NA	NA	−0.41	2014	J	5	C
Wang Kai et al.	616	0.279	NA	NA	2024	J	5	C
Wang Kai et al.	616	NA	−0.514	NA	2024	J	5	C
Wang Kai et al.	616	NA	NA	−0.187	2024	J	5	C
Wang Lingzhi et al.	303	0.58	NA	NA	2022	J	5	C
Xiao Tong	208	NA	NA	−0.25	2022	J	5	C
Yang Minfei et al.	286	0.357	NA	NA	2019	J	5	C
Yang Minli et al.	1,028	NA	−0.601	NA	2023	J	5	C
Yao Shili et al.	558	NA	−0.44	NA	2021	J	6	C
Wu Lianning et al.	690	NA	−0.444	NA	2024	J	5	C
Nese Ataman-Bor et al.	394	NA	NA	−0.557	2025	J	7	NC
Jinyu Li et al.	999	0.173	NA	NA	2025	J	6	C
Jinyu Li et al.	999	NA	−0.681	NA	2025	J	6	C
Jinyu Li et al.	999	NA	NA	−0.091	2025	J	6	C
Agbaria, Qutaiba et al.	500	NA	−0.49	NA	2021	J	7	NC
Brevers, Damien et al.	298	NA	−0.36	NA	2019	J	6	NC
Ding, Zhi-Chao et al.	1725	NA	−0.315	NA	2021	J	6	C
Dong, Junqiang et al.	1,272	NA	NA	−0.25	2024	J	7	C
Du, Zhihao et al.	950	NA	−0.54	NA	2022	J	6	C
Fu, Jiawei et al.	1,569	0.39	NA	NA	2021	J	6	C
Gülay Taşdemir et al.	536	NA	−0.13	NA	2024	J	6	NC
Haleem, Maryam et al.	562	0.32	NA	NA	2023	J	6	NC
Han, Lei et al.	543	NA	−0.529	NA	2017	J	5	C
Juslin, Jacob	205	NA	NA	−0.219	2020	J	5	NC
Kim, Jinha et al.	377	NA	−0.141	NA	2017	J	5	NC
kim, Myung Sig et al.	529	NA	NA	−0.21	2014	J	5	NC
Koc, Hayri et al.	866	NA	−0.29	NA	2023	J	5	NC
Li, Rui et al.	813	NA	NA	−0.22	2024	J	6	C
Li, Xinwei et al.	1,078	NA	−0.46	NA	2021	J	5	C
Li, Xinwei et al.	3,189	NA	−0.57	NA	2022	J	5	C
Liang, Sancai et al.	494	0.51	NA	NA	2022	J	6	C
Liu, Chong et al.	370	NA	−0.32	NA	2023	J	6	C
Liu, Yang et al.	697	NA	−0.55	NA	2024	J	6	C
Mak, Kwok Kei et al.	837	NA	−0.32	NA	2018	J	6	NC
Özdemir, Yalçın et al.	648	NA	−0.32	NA	2014	J	5	NC
Öztürk, Ayfer et al.	1,028	NA	NA	−0.498	2021	J	6	NC
Pan, Weigang et al.	526	NA	−0.599	NA	2023	J	5	C
Peng, Yu et al.	628	NA	−0.43	NA	2022	J	5	C
Qi, Wei et al.	483	NA	−0.53	NA	2024	J	5	C
Shen, Xuwei et al.	861	NA	−0.61	NA	2023	J	6	C
Sookbin, Im et al.	400	0.32	NA	NA	2014	J	6	NC
Sun, Yan et al.	471	NA	−0.349	NA	2025	J	7	C
Tafoya, Silvia Aracely et al.	32	0.56	NA	NA	2023	J	5	NC
Wang, Fang	413	0.452	NA	NA	2025	J	6	C
Wu, Jinlong et al.	590	NA	NA	−0.31	2024	J	7	C
Wu, Shujie et al.	560	0.362	NA	NA	2024	J	6	C
Wu, Shujie et al.	560	NA	−0.453	NA	2024	J	6	C
Wu, Shujie et al.	560	NA	NA	−0.36	2024	J	6	C
Yang, Yue-Di et al.	765	0.474	NA	NA	2024	J	6	C
Yin, Zhonggen et al.	2,274	NA	−0.414	NA	2024	J	7	C
Yu, Haoran et al.	473	NA	−0.006	NA	2024	J	6	C
Zhang, Anqi et al.	397	NA	−0.55	NA	2022	J	6	C
Zhang, Zhihao et al.	747	NA	−0.591	NA	2022	J	6	C
Zhao, Zitong et al.	257	NA	NA	−0.14	2022	J	6	C
Zheng, Wenkai et al.	900	NA	−0.468	NA	2024	J	6	C
Kang Da-jeong et al.	236	NA	−0.47	NA	2017	J	5	NC
Gülay Taşdemir et al.	536	NA	NA	−0.15	2024	J	6	NC
Wang, Fang	413	NA	NA	−0.408	2025	J	6	C

s_r is the correlation coefficient between self-control and psychological resilience; r_a is the correlation coefficient between psychological resilience and digital addiction; s_a is the correlation coefficient between self-control and digital addiction. D refers to degree theses, and J refers to published journal articles. C represents the China region, NC represents the non-China region.

### Main effect test

**Figure 2:**
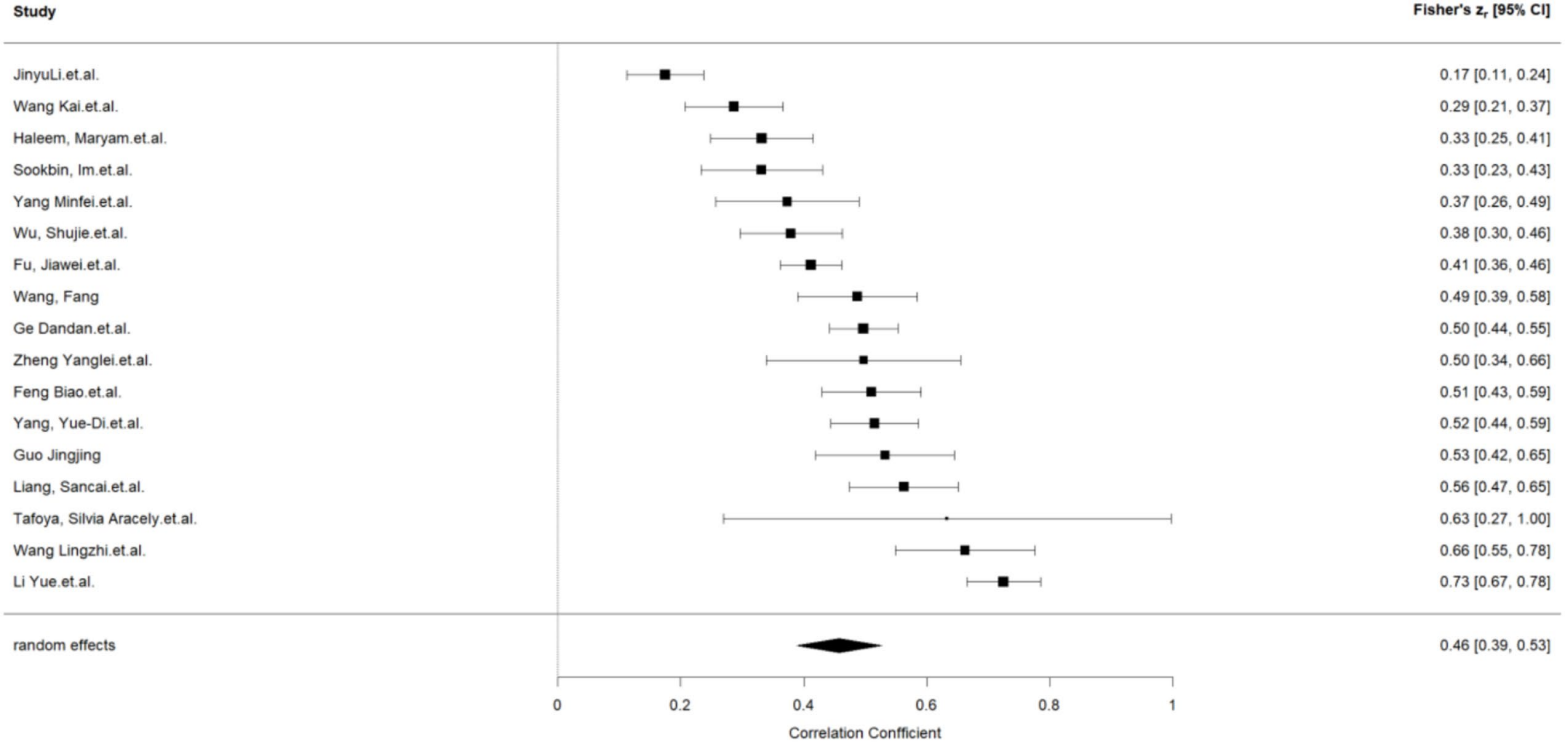
Self-control and psychological resilience.

**Figure 3:**
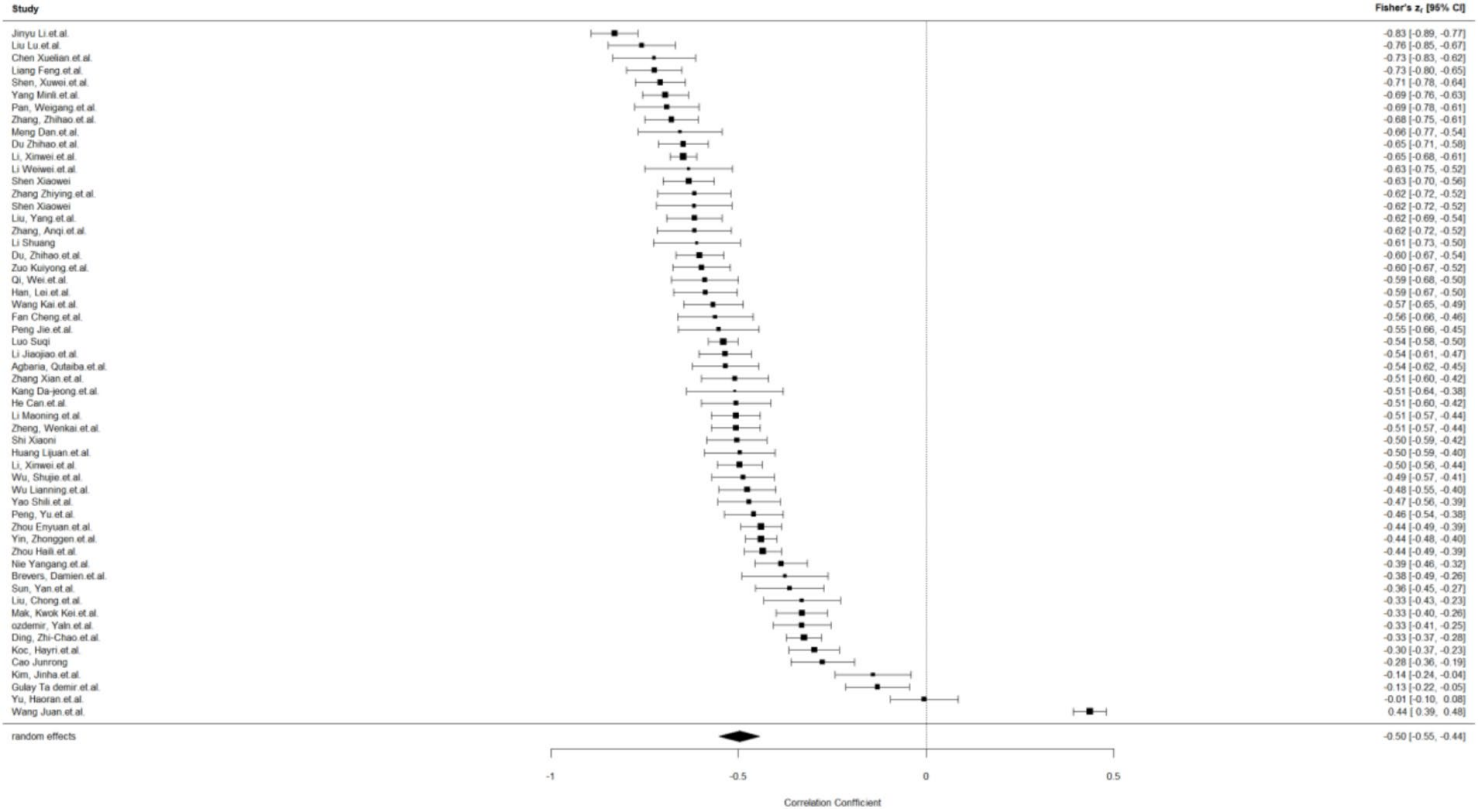
Self-control and digital addiction.

**Figure 4:**
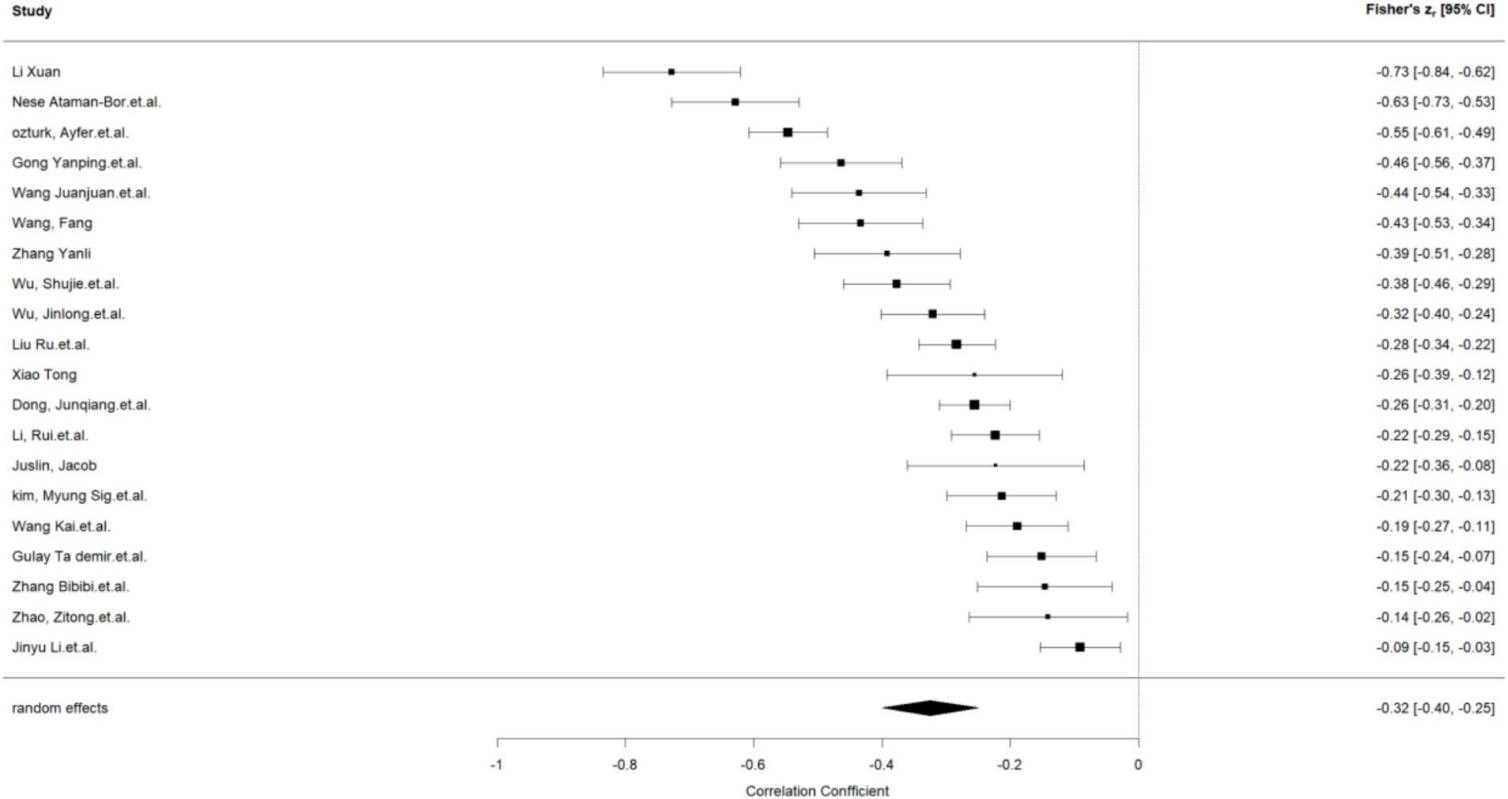
Psychological resilience and digital addiction.

This work performed a meta-analysis of the correlation coefficients for each variable pair and developed a random effects model to enhance the creation of the meta-analysis structural model. The findings indicated a moderate positive correlation between self-control and psychological resilience (*r* = 0.43, 95% CI: 0.37–0.48, *p* < 0.001); a negative correlation between self-control and digital addiction (*r* = −0.46, 95% CI: −0.50 −0.42, *p* < 0.001). and a moderate negative correlation between psychological resilience and digital addiction (*r* = −0.31, 95% CI: −0.38 −0.24, *p* < 0.001); The effect estimates of the univariate analyses exhibit minor discrepancies compared to those of the multivariate studies within the structural equation model (see Figures 2–4).

### Publication bias

**Figure 5:**
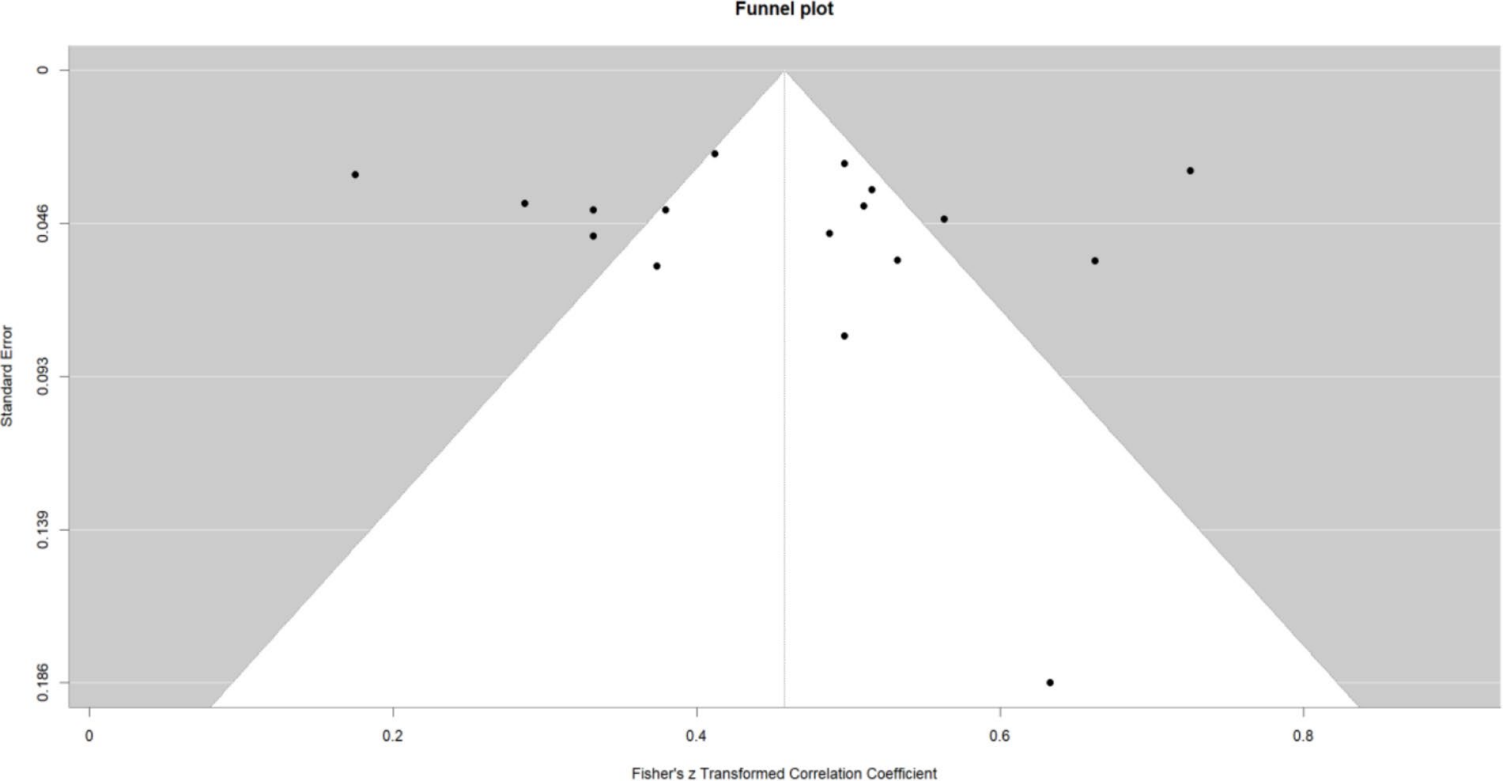
Self-control and psychological resilience funnel plot.

**Figure 6:**
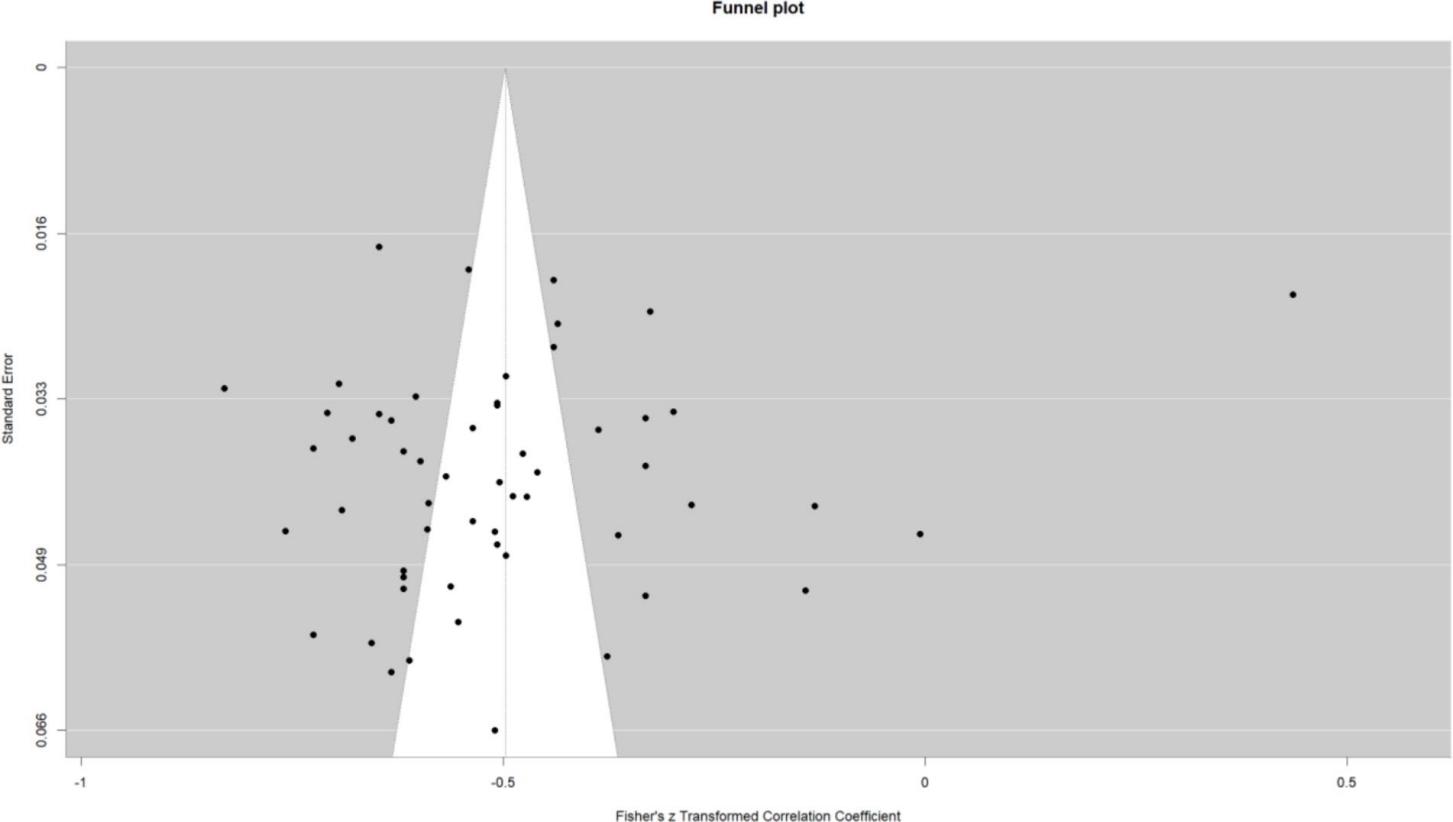
Self-control and digital addiction funnel plot.

**Figure 7:**
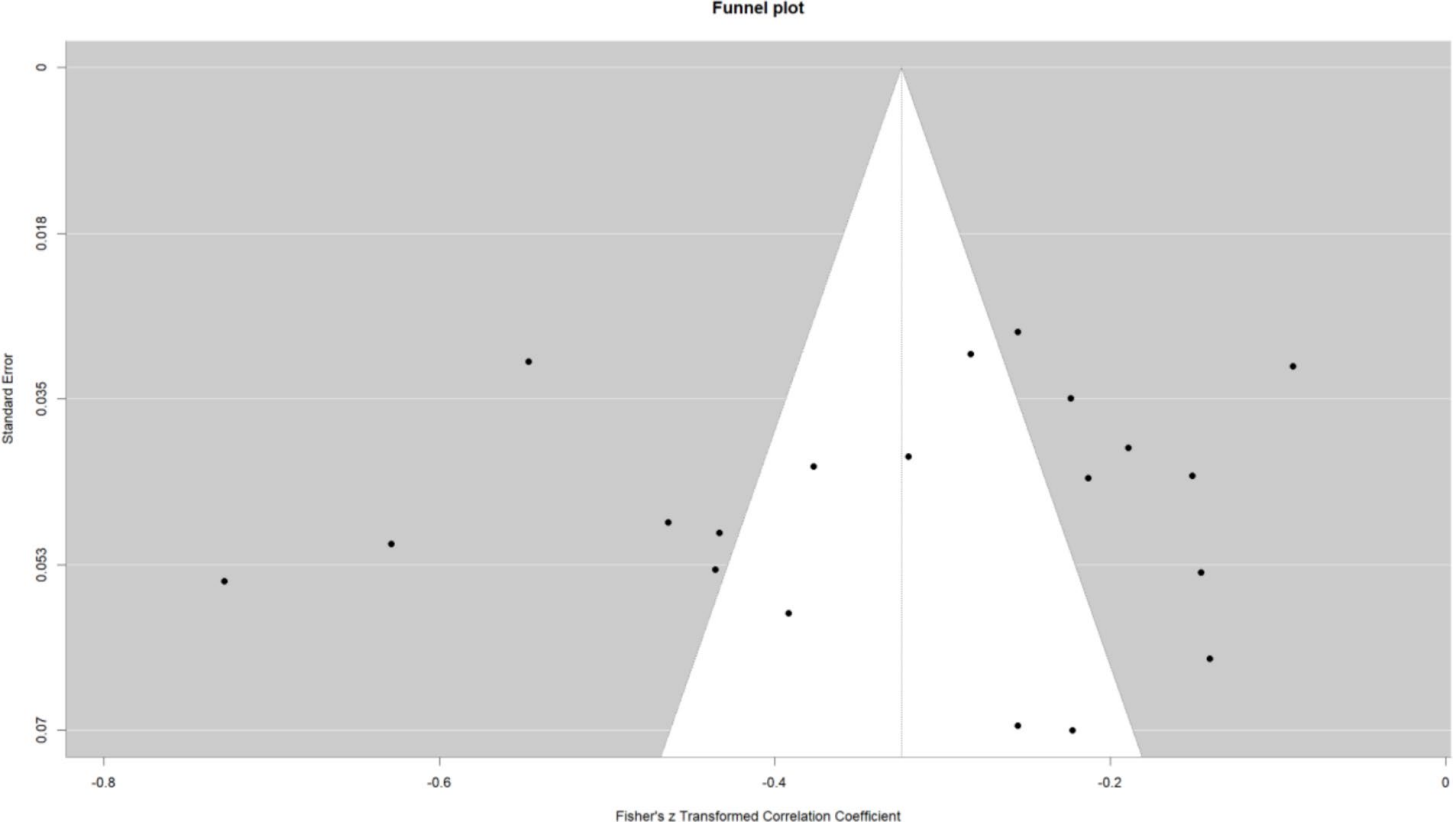
Psychological resilience and digital addiction funnel plot.

Publication bias studies on the associations between variable pairs reveal a notable degree of dispersion in the funnel plot. *Egger’s* test was performed to further validate the results. The findings indicated that the *p*-values for the correlations between self-control and psychological resilience (*p* = 0.37), self-control and digital addiction (*p* = 0.32) and psychological resilience and digital addiction (*p* = 0.71), were as follows. All these *p* > 0.05, signifying the absence of publication bias among the three variables. Statistically, no significant difference exists, and symmetry also shows no significant difference (*p* > 0.05; see Figure 5–7).

### Structural equation modeling stage 1: aggregating correlations

A meta-analysis of the initial phase of TESEM demonstrated considerable heterogeneity across all analyses, with notable variations among trials (Q = 3575.93, df = 90, *p* < 0.001). The particular results are as follows:

Self-control exhibited a correlation with psychological resilience (*I*^2^ = 92.08%, *p* < 0.001); self-control was connected with digital addiction (*I*^2^ = 96.89%, *p* < 0.001); psychological resilience correlated with digital addiction (*I*^2^ = 94.00%, *p* < 0.001), and The relationships between the variables are as follows: The correlation between self-control and psychological resilience is *r* = 0.42 (95% CI: 0.37, 0.48); the correlation between self-control and digital addiction is *r* = −0.45 (95%CI: −0.50, −0.40); the correlation between psychological resilience and digital addiction is *r* = −0.31 (95%CI: −0.37, −0.24; See Table 2).

**Table 2:** Flow diagram describing the process of study selection.

Path	SE	Weighted Average Correlation		95% CI		*I^2^*	*p*
		r	z	LL	UL		
Self-control and psychological resilience	0.03	0.42	15.06	0.37	0.48	92.08%	*p* < 0.001
Self-control and digital addiction	0.02	−0.45	−19.15	−0.50	−0.40	96.89%	*p* < 0.001
Psychological resilience and digital addiction	0.03	−0.31	−9.50	−0.37	−0.24	94.00%	*p* < 0.001

### Structural equation modeling stage 2: mediation model

**Figure 8:**
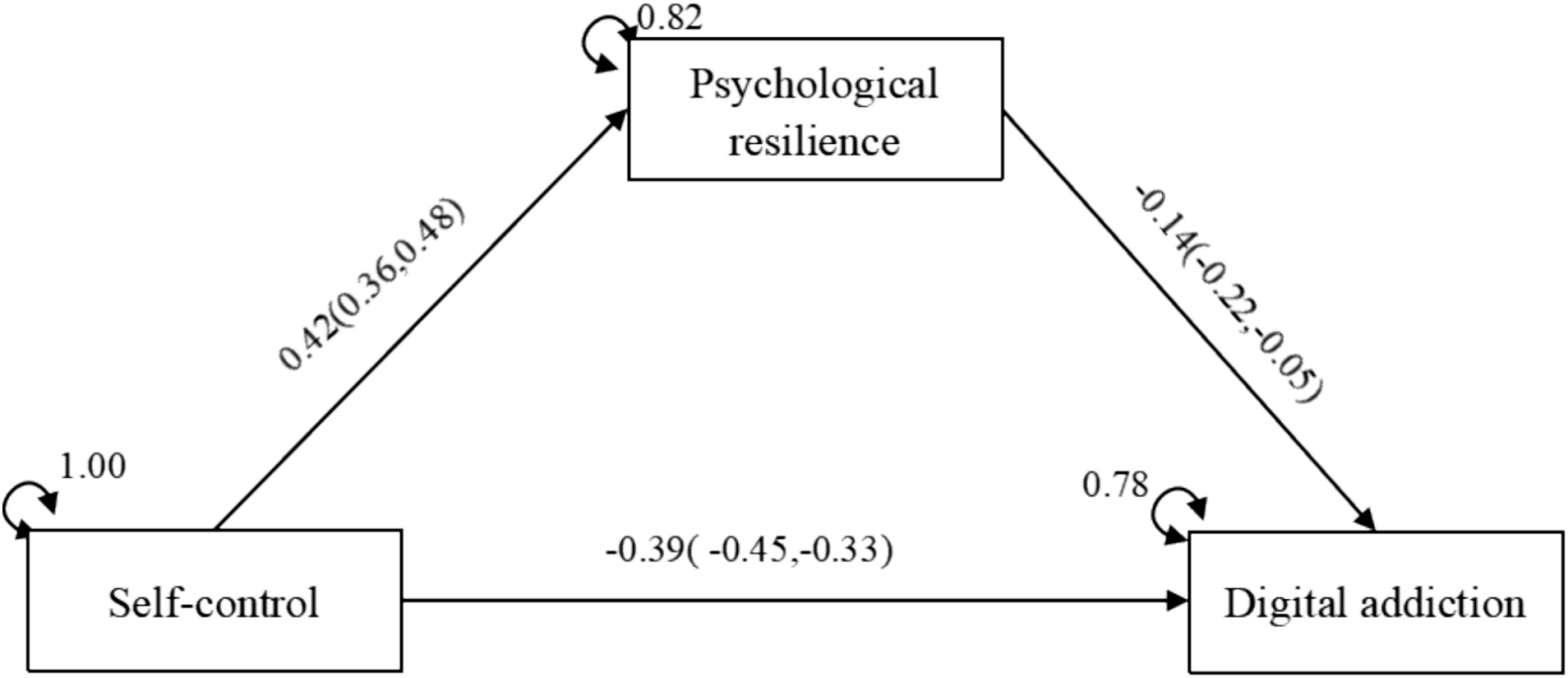
Intermediate model.

The analysis of the second stage of TESEM indicated that certain mediation models achieved saturation (*χ*^2^ = 0.00; CFI = 1.000; RMSEA = 0.000). Mediation effects were evaluated by analyzing the significance of both direct and indirect effects. In the partial mediation model, self-control demonstrated a direct negative effect on digital addiction (b = −0.39, 95% CI:-0.45, −0.33). Additionally, through the mediation of psychological resilience, self-control exhibited an indirect effect on digital addiction (b = −0.06, 95% CI:-0.09, −0.02). The findings support the four previously proposed research hypotheses: Hypothesis 1 indicates that self-control positively predicts psychological resilience; Hypothesis 2 suggests that self-control directly and negatively predicts digital addiction; Hypothesis 3 posits that psychological resilience negatively predicts digital addiction; and Hypothesis 4 asserts that psychological resilience partially mediates the relationship between self-control and digital addiction. All hypotheses were fully validated (see Figure 8).

### Moderate effect analysis

Subgroup analyses were performed according to article genre, quality, and area. The subsequent table enumerates the meta-analysis estimates of the moderating effects (see Table 3).

**Table 3:** Direct and indirect in the meta-analytic mediation models.

Overall results and subgroup analysis	k	Indirect effects	Direct effects
All studies	93	−0.06 (−0.09, −0.02)	−0.39 (−0.45, −0.33)
Subgroup analysis			
Low score group	76	−0.06 (−0.09, −0.02)	−0.39 (−0.46, −0.31)
High score group	17	−0.07 (−0.15, 0.02)	−0.41 (−0.54, −0.29)
Chinese samples	77	−0.05 (−0.09, −0.01)	−0.42 (−0.49, −0.35)
Non-Chinese samples	16	−0.08 (−0.14, −0.03)	−0.23 (−0.34, −0.12)
Thesis	7	−0.09 (−0.16, −0.02)	−0.37 (−0.51, −0.24)
Journal article	86	−0.06 (−0.09, −0.02)	−0.39 (−0.46, −0.32)

Participants were categorized into high- and low-scoring groups according to article quality ratings. The results indicated that the high-scoring group demonstrated an indirect effect of (b = −0.07, 95% CI: −0.15, 0.02) and a direct effect of (b = −0.41, 95% CI: −0.54, −0.29). In contrast, the low-quality group exhibited an indirect effect of (b = −0.06, 95% CI: −0.09, −0.02) and a direct effect of (b = −0.39, 95% CI: −0.46, −0.31). Upon analysis of moderating effects, no significant moderating effect of article quality was identified (*χ^2^* = 6.400, df = 3.0, *p* > 0.05).

The analysis was primarily categorized into China and non-China regions. The findings for the China region reveal an indirect effect of (b = −0.05, 95% CI: −0.09, −0.01) and a direct effect of (b = −0.42, 95% CI: −0.49, −0.35). The indirect effect for the non-China region was (b = −0.08, 95% CI: −0.14, −0.03), while the direct effect was (b = −0.23, 95% CI: −0.34, −0.12). The moderation analysis indicated a significant moderating effect of region (*χ^2^* = 16.869, df = 3.0, *p* < 0.001).

The analysis primarily differentiated between dissertations and journal articles. The results showed that for dissertations, the indirect effect was (b = −0.09, 95% CI: −0.16, −0.02) and the direct effect was (b = −0.37, 95% CI: −0.51, −0.24). The indirect effect for journal articles was (b = −0.06, 95% CI: −0.09, −0.02), while the direct effect was (b = −0.39, 95% CI: −0.46, −0.32). The analysis of moderating effects indicated that article type did not have a moderating effect (*χ^2^* = 3.288, df = 3.0, *p* > 0.05).

## Discussion

This study discovered that self-control inversely predicts digital addiction in college students, along with the hypothesis of limited self-control. Self-control is a finite psychological resource; when individuals exert considerable self-control, their psychological resources markedly deplete, resulting in heightened consumption of digital media (Chen et al., 2024). College students with reduced self-control exhibit a more rapid exhaustion of this psychological resource when managing academic stress. As a result, individuals engage in activities such as browsing short films or utilizing social media, ultimately developing an addiction to the internet.

Structural equation modeling demonstrates that psychological resilience regulates the association between self-control and digital addiction. The fundamental mechanism of this mediating route involves self-control behaviors that bolster psychological resilience via the accumulation of successful experiences. Individuals possessing elevated self-control may proficiently suppress cravings for immediate implementation when confronted with academic stress or social challenges. They instead employ cognitive reappraisal or problem-focused tactics, diminishing escapist dependence on digital media and thereby enhancing psychological resilience. College students possessing good self-control cultivate resilience by engaging in long-term goal-oriented behaviors, such as devising and implementing academic strategies or participating in research initiatives. Conquering procrastination and managing competition in these pursuits cultivates experience that markedly improves psychological resilience (Feng et al., 2022). Moreover, when confronted with loneliness, highly self-disciplined college students are inclined to participate in offline social activities instead of engaging in internet connections. Effectively obtaining emotional support and mitigating negative emotions through real-world contacts represents a vital avenue for improving social efficacy and psychological resilience (Chen et al., 2024). Ultimately, improved psychological resilience, with its fundamental abilities like emotional regulation and problem-solving, diminishes the likelihood of digital addiction.

Subgroup analysis indicated that regional samples dramatically altered the relationship model among self-control, psychological resilience, and digital addiction, further highlighting the substantial impact of cultural values on psychological mechanisms. This effect is strongly associated with long-term goal orientation (Hofstede et al., 2008). The long-term goal orientation intrinsic to Chinese culture generates distinct societal forces that intensify the direct impacts of self-control. In China’s higher education system, competitive pressures, including postgraduate entrance examinations and employment requirements, influence the whole university experience, necessitating that students uphold academic achievement by rigorous self-discipline. A midst significant stress, college students are increasingly inclined to view digital media as an impediment to their long-term objectives, thereby reducing consumption to maintain academic success. Moreover, familial characteristics significantly contribute to elucidating this occurrence. In China, parents have elevated expectations regarding their children’s academic performance (TONG Xing, 2025). This may drive college students to perceive self-control as a method of meeting familial obligations. This external motive amplifies the direct inhibitory impact of self-control on digital addiction.

### Strengths, limitations, and future research directions

This meta-analysis utilized structural equation modeling to clarify the mediating effect of psychological resilience on the relationship between self-control and digital addiction in college students. The findings not only enhance limited self-control theory but also critically integrate research discoveries with established behavioral addiction intervention models and resilience-building intervention models, thereby enriching the theoretical framework of multi-model integration.

This study presents information regarding the correlation between self-control, psychological resilience, and digital addiction among college students; nonetheless, it has significant limitations: The operational definitions of digital addiction differed among studies, including several forms such as mobile phone addiction and internet addiction, leading to significant model heterogeneity that may mask the distinct psychological mechanisms associated with different types of digital addiction. Future study may categorize digital addiction by medium or function, utilizing meta-analyses or empirical investigations to investigate varying pathways. Meta-analyses utilizing cross-sectional observational data can only confirm associative relationships between variables, rather than determine causal directionality. Future research should utilize longitudinal tracking to investigate whether self-control and psychological resilience forecast later digital addiction. The sample makeup demonstrates regional bias, featuring an excessively large Chinese sample and comparatively smaller non-Chinese samples. The examination of regional moderation effects predominantly depends on Chinese data, leading to diminished explanatory ability for psychological mechanisms in non-Chinese populations. This substantially affects the universality of conclusions, requiring prudence when extrapolating findings about regional moderating effects. Future study should encompass college student samples from many cultural backgrounds, including Europe, America, Africa, and Latin America, to analyze the differing impacts of self-control and psychological resilience across cultural contexts.

### Impact on policies and schools

This study determined that psychological resilience modulates the association between self-control and digital addiction, thereby elucidating the connection among these variables. These findings offer significant insights for policymakers and professionals within the education sector.

A coordinated support system for digital addiction intervention should be built in policy creation, incorporating the education, health, and cyberspace administration sectors. Responsibilities must be explicitly delineated: Educational departments spearhead campus prevention initiatives and risk assessments for digital addiction, incorporating digital literacy into campus administration; Health departments furnish professional psychological assessment instruments and intervention resources to assist at-risk students; Cyberspace administration authorities can concentrate on regulating digital service establishments adjacent to universities to mitigate the incursion of detrimental digital content. Simultaneously, specific research funds must be allocated to assist universities in executing research and practices related to digital addiction interventions grounded in this theoretical paradigm, thereby improving the scientific integrity of intervention strategies.

Educational institutions can systematically improve students’ self-discipline and psychological resilience through structured curricula and training programs. Specialized courses should be designed, including effective training techniques to assist students in mastering essential skills such as emotional regulation and behavioral control. These should be supplemented by various practical activities, like group counseling and resilience-building exercises, to enhance psychological resilience in real-world contexts. Furthermore, environmental strategies must be instituted to mitigate triggers of digital addiction. Mobile phone storage solutions can be implemented in educational settings such as libraries, academic buildings, and study rooms to redirect students from digital distractions during study sessions. Simultaneously, enhancing offline campus cultural activities—such as instituting weekly academic salons and cultural or sports competitions—can fortify students’ real-world social relationships and self-esteem, therefore diminishing their need on digital media.

## Conclusion

This study systematically synthesized research on the interplay between self-control, psychological resilience, and digital addiction among college students using a meta-analytic structural equation model, elucidating the following relationships: Self-control and psychological resilience exhibit a significant positive correlation; self-control and digital addiction demonstrate a significant negative correlation; and psychological resilience partially mediates the relationship between self-control and digital addiction. Self-control is the fundamental psychological resource for resisting digital addiction, functioning directly by suppressing immediate impulses and indirectly by enhancing psychological resilience to create a long-term protective mechanism. College students exhibiting high self-control are more inclined to develop psychological resilience through goal-directed actions, consequently diminishing their dependence on digital media as a means of escapism. The analysis of the moderation effect demonstrated that regional factors significantly influence the mediation model.

## Data Availability

The datasets presented in this study can be found in online repositories. The names of the repository/repositories and accession number(s) can be found at: https://www.scidb.cn/anonymous/YXlBbmVp.
