# Correction: The relationships among self-control, psychological resilience, and digital addiction in college students: a meta-analytic structural equation modeling

**DOI:** 10.3389/fpsyg.2025.1712264

**Published:** 2025-10-16

**Authors:** Ziyang Zhao, Yaozhong Zhang, Yongqiang Ma, Azeem Obaid, Haidong Zhu

**Affiliations:** ^1^Normal College of Shihezi University, Xinjiang, China; ^2^Psychological Application Research Center, Shihezi University, Shihezi, China

**Keywords:** self-control, psychological resilience, digital addiction, meta-analysis, mediating effect

The order of authors in the author list of the published paper was erroneously given as:

[Ziyang Zhao^1^†, Yaozhong Zhang^1^, Haidong Zhu^1^^, 2^^*^, Yongqiang Ma^1^†, Azeem Obaid^1^]

The correct author list reads:

[Ziyang Zhao^1^†, Yaozhong Zhang^1^†, Yongqiang Ma^1^, Azeem Obaid^1^, Haidong Zhu^1^^, 2^^*^]

Author [Yongqiang Ma] was erroneously assigned as equal contributing author.

Author [Yaozhong Zhang] was erroneously omitted as equal contributing author.

The original version of this article has been updated.

